# Male‐biased dispersal and the potential impact of human‐induced habitat modifications on the Neotropical bat *Trachops cirrhosus*

**DOI:** 10.1002/ece3.4161

**Published:** 2018-05-15

**Authors:** Tanja K. Halczok, Stefan D. Brändel, Victoria Flores, Sébastien J. Puechmaille, Marco Tschapka, Rachel A. Page, Gerald Kerth

**Affiliations:** ^1^ Zoological Institute and Museum Greifswald University Greifswald Germany; ^2^ Smithsonian Tropical Research Institute Balboa, Ancón Republic of Panamá; ^3^ Institute of Evolutionary Ecology and Conservation Genomics University of Ulm Ulm Germany; ^4^ Committee on Evolutionary Biology University of Chicago Chicago Illinois

**Keywords:** gene flow, Neotropics, population genetics, sex‐biased dispersal, *Trachops cirrhosus*

## Abstract

Gene flow, maintained through natal dispersal and subsequent mating events, is one of the most important processes in both ecology and population genetics. Among mammalian populations, gene flow is strongly affected by a variety of factors, including the species’ ability to disperse, and the composition of the environment which can limit dispersal. Information on dispersal patterns is thus crucial both for conservation management and for understanding the social system of a species. We used 16 polymorphic nuclear microsatellite loci in addition to mitochondrial DNA sequences (1.61 kbp) to analyse the population structure and the sex‐specific pattern of natal dispersal in the frog‐eating fringe‐lipped bat, *Trachops cirrhosus*, in Central Panama. Our study revealed that—unlike most of the few other investigated Neotropical bats—gene flow in this species is mostly male‐mediated. Nevertheless, distinct genetic clusters occur in both sexes. In particular, the presence of genetic differentiation in the dataset only consisting of the dispersing sex (males) indicates that gene flow is impeded within our study area. Our data are in line with the Panama Canal in connection with the widening of the Río Chagres during the canal construction acting as a recent barrier to gene flow. The sensitivity of *T. cirrhosus* to human‐induced habitat modifications is further indicated by an extremely low capture success in highly fragmented areas. Taken together, our genetic and capture data provide evidence for this species to be classified as less mobile and thus vulnerable to habitat change, information that is important for conservation management.

## 1 INTRODUCTION

The amount of gene flow is an important determinant for genetic differentiation among populations (e.g., Slatkin, 1985). As it can influence effective population size, genetic diversity, local adaptation, and ultimately speciation, gene flow is one of the most important processes in both population genetics and ecology (e.g., Bohonak, 1999). In the absence of this transfer of genetic material between populations, a combination of mutations and genetic drift cause genetic divergence of populations. Gene flow between populations can be maintained by dispersal and subsequent mating events (Freeland, Kirk, & Petersen, 2011b). Generally, life history, behavioral, morphological, and habitat‐associated traits all contribute to a species’ dispersal ability (Bohonak, 1999; Bonte et al., 2012). Therefore, dispersal can be influenced by a variety of factors, including the availability of suitable mating partners and resources, and the occurrence of pathogens, parasites, and predators (Freeland et al., 2011b).

Moreover, in addition to a species’ general ability and propensity to move, dispersal and thus gene flow can also be impeded by the composition of the environment (Freeland et al., 2011b). In a landscape where physical barriers to dispersal occur, for example, rivers or mountain ranges, restricted connectivity of habitats may lead to population genetic differentiation (e.g., Manel, Schwartz, Luikart, & Taberlet, 2003). Another major factor that impedes dispersal and contributes to isolation and subdivision of populations is human‐induced habitat fragmentation (e.g., Rudel, Defries, Asner, & Laurance, 2009). Forest fragments often are embedded in a matrix of inhospitable habitat types causing isolation and subdivision of animal populations (e.g., Watling & Donnelly, 2006). Consequently, genetic drift in combination with reduced gene flow in fragmented landscapes may result in an increase of genetic differentiation and a loss of genetic diversity (e.g., Fahrig, 2003).

Even though bats are generally considered to be able to cross open areas due to their high mobility, different species‐specific reactions to habitat fragmentation have been reported (e.g., Avila‐Cabadilla et al., 2012; Ferreira et al., 2017; Kerth & Melber, 2009). Depending on the matrix the remaining habitat patches are located in, bats can be more tolerant to habitat modifications compared to other animals due to their capacity to fly and their ability to exploit resources that are patchy in time and space (e.g., Bernard & Fenton, 2007; O'Donnell, Richter, Dool, Monks, & Kerth, 2016). However, in fragmented areas with an unfavorable matrix, bats can be sensitive to the modified habitats or forest edges (e.g., Meyer, Kalko, & Kerth, 2009; Ripperger, Tschapka, Kalko, Rodriguez‐Herrera, & Mayer, 2013). For many forest‐dwelling bat species, open water seems to be one of the least favorable types of matrix, as it provides no protection from potential predators and offers limited resources (e.g., Albrecht, Meyer, & Kalko, 2007).

Among mammalian populations, gene flow is often strongly affected by sex‐biased dispersal (Perrin & Mazalov, 2000). In particular, many species of colonially breeding animals exhibit sex‐biased behaviors (Greenwood, 1980). While in mammals, stronger philopatry to their natal area is typically shown by females, whereas males often disperse when reaching maturity, the opposite pattern has been described for birds (Dobson, 1982; Greenwood, 1980). The main evolutionary forces suggested to shape sex‐specific dispersal patterns include kin cooperation and the avoidance of inbreeding, local mate competition, and local resource competition (see Lawson Handley & Perrin, 2007 for a review).

As bats are small, highly mobile, and nocturnal, their dispersal can be challenging to monitor using radio telemetry and capture‐mark‐recapture methods (e.g., Petit & Mayer, 1999). However, population genetics can shed light into mating and dispersal behavior of bats. Providing a complementary approach to traditional field techniques, genetic approaches allow us to estimate the degree of population structuring and, therefore, provide cost efficient, relatively noninvasive methods for surveying the spatial structure of mammalian populations (e.g., Frantz, Do Linh San, Pope, & Burke, 2010). As the degree of genetic differentiation between and within subpopulations is affected by dispersal, philopatry, and the mating system, understanding population structure can provide insights into the social organization of a species (e.g., Burland & Wilmer, 2001). Additionally, different markers can be used to assess different aspects of the population genetic composition of a population. Differences in mutation rate between nuclear microsatellite loci and mitochondrial DNA (mtDNA) allow for a different resolution in terms of time scale. Whereas microsatellites provide excellent resolution to understand contemporary gene flow (e.g., Angers & Bernatchez, 1998), the much lower mutation rates of coding regions of mtDNA reflect rather historical signals (Avise et al., 1987). Moreover, as the variability of bi‐parentally and uni‐parentally inherited loci may be affected differently, for example, by the presence of sex‐biased dispersal, it is often informative to use both mitochondrial and nuclear molecular markers (e.g., Castella, Ruedi, & Excoffier, 2001; Kerth, Mayer, & Petit, 2002).

The fringe‐lipped bat, *Trachops cirrhosus* (Phyllostomidae), is a Neotropical animalivorous species that occurs from southern Mexico to southern Brazil (Cramer, Willig, & Jones, 2001). While it is widespread in lowland forest, this species is rather rare in agricultural areas and at higher elevations (Cramer et al., 2001). It roosts in caves, hollow trees, road culverts, and buildings in groups of up to 50 individuals (Hall & Dalquest, 1963), where both sexes can be encountered roosting together (Nowak, 1999). *Trachops cirrhosus* hunts frogs and various insects (Cramer et al., 2001; Tuttle & Ryan, 1981) and its relatively small foraging grounds (3–12 ha) are typically located 200 m to 1.6 km from its roost (Jones, Hamsch, Page, Kalko, & O'Mara, 2017; Kalko, Friemel, Handley, & Schnitzler, 1999). Although numerous studies have been conducted on the predatory preferences and the foraging behavior of *T. cirrhosus* (reviewed in Page & Jones, 2016), very little is known about dispersal and population dynamics in this species. Generally, the mating system of most leaf‐nosed bats (Phyllostomidae) is not known and is likewise unknown for *T. cirrhosus* (McCracken & Wilkinson, 2000). Furthermore, information is scarce about the effects of environmental disturbances on the population genetic structure of insectivorous or carnivorous bats in the Neotropics in general (Cunto & Bernard, 2012; Fenton et al., 1992).

**Figure 1:**
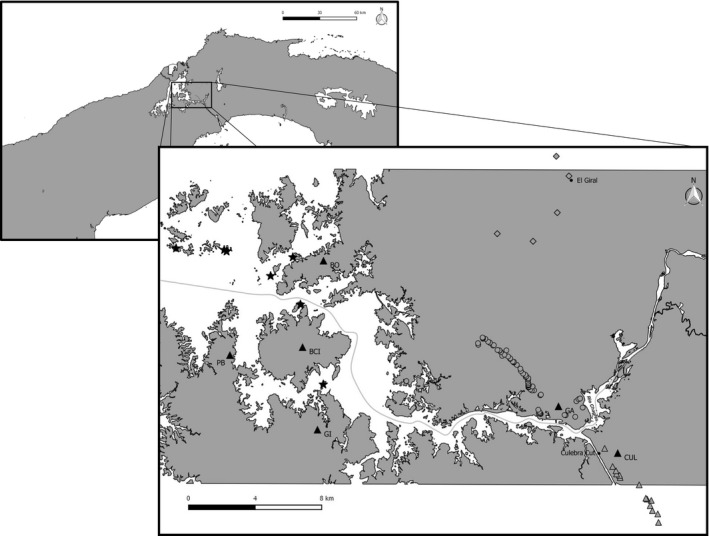
The study area and sampling sites of *Trachops cirrhosus* in Central Panama. The filled triangular markings represent the different sampling sites Barro Colorado Island (BCI); Peña Blanca (PB), Bohio (BO), Gigante (GI), Culebra Cut (CUL), and Gamboa (GA). As GA and CUL both consist of various netting sites, those are indicated by open circles for GA and open triangles for CUL. The stars represent all islands were sampling efforts were undertaken (*I*) and the diamonds represent those netting sites summarized as *A*. The gray line roughly represents the former route of Río Chagres before the construction of the Panama Canal (Shepherd, 1911)

While little more than 100 years ago the only potential barrier to gene flow for *T. cirrhosus* in our study area in Central Panama might have been the Río Chagres (Figure 1), the construction of the Panama Canal has caused additional large‐scale fragmentation to its habitat. Most lowland forest has been flooded through the damming of the Río Chagres between 1910 and 1914, which caused former hilltops to become isolated islands surrounded by a matrix of water (Albrecht et al., 2007). Over 200 such islands covered with semideciduous, lowland tropical moist forest exists within the Panama Canal varying in size and degree of isolation, the largest being Barro Colorado Island (BCI) with 1,560 ha (Leigh, 1999). In 1999, Kalko et al. predicted that habitat alterations, particularly fragmentation and isolation of forested areas, would negatively affect populations of *T. cirrhosus*. This study indicated that the relatively sedentary foraging behavior of *T. cirrhosus*, reflected in its wing morphology and its use of small foraging areas, makes this bat species vulnerable to habitat changes.

Therefore, the aim of our study was to use population genetic tools to assess patterns of gene flow between distinct populations of *T. cirrhosus* in this highly fragmented landscape in Central Panama and to determine whether gene flow is driven by sex‐specific natal dispersal in this species. Based on the available information of the species’ biology, we hypothesized that populations of *T. cirrhosus* within our study area would be genetically differentiated and expected to find evidence for the impact of habitat fragmentation on the genetic structure of populations of this species. However, for the natal dispersal pattern of *T. cirrhosus* we could not make a clear prediction: even though female philopatry has been hypothesized to be the strategy most beneficial for mammals, bats in the Neotropics have been shown to display various patterns of natal dispersal (e.g., Dechmann, Kalko, & Kerth, 2007; Wilkinson, 1985).

## 2 MATERIAL AND METHODS

### 2.1 Study area and sample collection

Bats were captured over an eleven‐year period, from 2005 to 2016, using mist nets (Avinet, Dryden NY, USA and Ecotone, Gdynia, Poland), set in the forest, along streams, near small ponds, and at known roosts. Wing tissue samples (4 mm ø wing punch) were collected and stored in >95% ethanol until DNA extraction.

Bats of both sexes were captured and juveniles were identified by the presence of epiphyseal gaps in the phalanges (Brunet‐Rossini & Wilkinson, 2009). All sampling protocols followed guidelines approved by the American Society of Mammalogists for capture, handling, and care of mammals (Sikes, 2016) and were conducted in accordance with the standards of the Smithsonian Tropical Research Institute (STRI) Institutional Animal Care and Use Committee (IACUC; 07113001, 04113002, 2007‐14‐06‐15‐07, 20100816‐1012‐16, 2014‐0101‐2017, 2013‐0401‐2016, 2016‐0627‐2019). All research was licensed and approved by the government of Panama (ANAM and MiAmbiente permits: SE/A‐43‐07, SE/A‐91‐09, SE/A‐95‐10, SE/A‐6‐11, SE/A‐46‐11, SE/A‐94‐11, SE/A‐58‐12, SE/A‐19‐13, SE/A‐75‐13, SE/A‐21‐14, SE/A‐69‐14, SE/A‐86‐14, SE/A 69‐15, SE/AH‐2‐16, SE/A‐28‐16).

Bats were captured on Barro Colorado Island (BCI, *N* = 41), on three peninsulas in the Barro Colorado National Monument (BCNM; namely Bohio (BO, *N* = 12), Gigante (GI, *N* = 9) and Peña Blanca (PB, *N* = 5), on the other side of the Río Chagres near the Culebra Cut (CUL, *N* = 42) and the areas surrounding the village of Gamboa situated at the shore of the Panama Canal (GA, *N* = 283), Central Panama (Figure 1). Some of these sampling sites pool many individual netting sites (see Figure 1 for details). Peña Blanca has been removed from the analyses of nuclear DNA (nucDNA) due to insufficient sample size; however, we nevertheless used it for the analyses of mtDNA. The maximum distance between our sampling sites was 27.3 km, between PB and CUL, and the minimum distance amounted to 2.2 km between the closest respective netting sites within GA and CUL.

Sampling efforts were also undertaken on various islands within the Panama Canal and in an area where forest patches are embedded in a matrix of agriculturally managed land close to the town El Giral (Figure 1) between 2013 and 2016 using an adapted standardized mist‐netting approach (Meyer & Kalko, 2008a). Despite the fact that the mist netting effort during this time was comparable to that of the other sampling sites, only one individual was encountered in each of these two highly fragmented areas, respectively. This insufficient sample size led to the exclusion of these two individuals from population genetic analyses.

### 2.2 Bat DNA extraction and amplification

Genomic DNA was extracted using an ammonium acetate precipitation method (Nicholls, Double, Rowell, & Magrath, 2000). Individuals were genotyped using 16 newly developed microsatellite markers (Table 1). For primer development, samples were prepared using a standard Illumina Nextera DNA kit (Illumina Inc.) and DNA sequencing was conducted using a MiSeq Benchtop Sequencer (Illumina Inc.) to create a library of paired‐end reads. Microsatellite repeats were identified using pal_finder_v0.02.04 (Castoe et al., 2012) and primers were designed around those regions using Primer3 version 2.3.6 (Untergasser et al., 2012). The 5′ ends of the reverse primers Tcir5 and Tcir12 were PIG‐tailed (Brownstein, Carpten, & Smith, 1996) with the sequence GTTTT to facilitate adenylation. These 16 autosomal microsatellite markers were amplified in two multiplex polymerase chain reactions (PCRs; Table S1, supplementary material). PCRs were carried out in 5 μl reaction volumes using the Qiagen Type‐it^®^ Microsatellite PCR Kit (Qiagen, Hilden, Germany). Each multiplex reaction contained 1× Qiagen Multiplex Master Mix and between 0.02 μmol/L and 1.10 μmol/L of each primer (Table S1). After drying 1 μl of DNA (approximately 10 ng/μl) for 15 min at 52°C in a 96‐well PCR plate (Thermo Fisher Scientific), multiplex reactions were performed using a touchdown program (64–58°C) due to differences in primer annealing temperatures. The PCR amplification was carried out in a Veriti Thermal Cycler (Applied Biosystems), with an initial 15‐min denaturation at 95°C, followed by denaturation at 94°C for 30 s, annealing at initially 64°C for 90 s and extension at 72°C for 90 s. The annealing temperature was reduced by 1°C per cycle for seven cycles and then kept at 58°C for the remaining 28 cycles. Final incubation occurred at 60°C for 30 min. PCR products were separated using an ABI 3130 Genetic Analyser (Applied Biosystems) together with the internal size standard genescan 500 liz (Applied Biosystems) and the data were analyzed using genemapper v 5.0 (Applied Biosystems).

**Table 1:** Characterization of the newly developed microsatellite loci for *Trachops cirrhosus*

Locus	Repeat motif	Size range (bp)	Multiplex; Label	GenBank accession no.	Primer sequences (5′–3′)
*Tcir2*	(GT)_21_	173–227	1; FAM	MF977666	F: AAACTTGTTACAGGCTCC
					R: CTTAATTAAACGTGACCC
*Tcir5*	(TTC)_19_	304–396	1; FAM	MF977667	F: AAGAGTAGAGAAATGGTGC
					R: CTCTTTAGAATCAATCAGC
*Tcir9*	(GATG)_10_	471–578	1; NED	MF977668	F: ATAGTTTAAGCTCACCTCC
					R: ATGAGAATATCTCTGGGG
*Tcir11*	(GT)_16_	82–114	1; NED	MF977669	F: GTGATCATCATATAATACGG
					R: TTAGTTCCTTTATGCTACC
*Tcir12*	(CT)_10_	263–328	2; VIC	MF977670	F: AGATTCAGACACCTACCC
					R: TCTGTACTCTTGAGCAGC
*Tcir13*	(GT)_15_	222–264	1; NED	MF977671	F: CATCATTTCTTCTGAACG
					R: TAAACACATTCGCATACC
*Tcir20*	(TTCC)_13_	315–378	2; FAM	MF977672	F: GCTGTAGTGTAGATTGTCC
					R: AAAGAAACACTATGAGCC
*Tcir22*	(TG)_10_	460–521	2; NED	MF977673	F: ATTCTCAACACATCATATCC
					R: ACATAGACAGTGCTCAGC
*Tcir24*	(ATC)_11_	168–218	2; NED	MF977674	F: ACAAACTCTTCTAATTGTGG
					R: ATGAACCTTTATTGACTACC
*Tcir25*	(CAA)_9_	369–406	2; PET	MF977675	F: ACAGCCTAACTATCTCTCC
					R: TTTTGAGAATAGAGGTCG
*Tcir26*	(ATGG)_11_	90–140	2; PET	MF977676	F: GACTCTGAGATCCCATGCTTGA
					R: ACCTTTTCCTTCACCTTCCCTC
*Tcir28*	(GAAG)_17_	277–349	1; VIC	MF977677	F: TTCTAAGTCCTCTAGCTACC
					R: AGGTAGCCAATGACTACC
*Tcir35*	(AATG)_8_	71–127	2; VIC	MF977678	F: TGATGTTTACTTCAGCCTGGC
					R: CCTCTGGAAGCCTTTGTTCG
*Tcir38*	(ATTT)_10_	263–324	2; NED	MF977679	F: GAATGAACACTGTCTCAGG
					R: ACTTGGACTAAAAGAGGC
*Tcir39*	(CCTC)_15_	335–394	2; VIC	MF977680	F: TCCAACTGACTGATAACC
					R: ACCATAAGTTTAGTCCAGG
*Tcir40*	(AAAC)_8_	473–556	1; PET	MF977681	F: ATACTGGCTATGTCATTACC
					R: CACTGTTCTTCTGTAACAGG

The following abbreviations are used: the observed fragment length range (Size range), in base pair (bp), the multiplex in which each marker was included (Multiplex) and which fluorescent label was used (Label) and both primer sequences (F, forward; R, reverse). All sequences have been deposited in the GenBank under the accession numbers provided.

For the mtDNA, the entire cytochrome *b*, tRNA threonine, tRNA proline, and parts of the control region of the D‐loop were amplified for a geographically representative subset of individuals (*N* = 53; *N*_CUL_ = 6, *N*_GA_ = 16, *N*_GI_ = 5, *N*_BCI_ = 15, *N*_BO_ = 6, *N*_PB_ = 5) using the primers mt‐DNA‐R3‐F and mt‐DNA‐F2‐R (Puechmaille et al., 2011) resulting in a 1,605 bp alignment. This PCR was carried out in a 25 μl reaction volume using the Qiagen Multiplex PCR Kit (Qiagen, Hilden, Germany), which contained 2 μl of DNA (approximately 10 ng/μl), 1× Qiagen Multiplex Master Mix and 0.2 μmol/L of each primer. PCR amplification was carried out in a 2720 Thermal Cycler (Applied Biosystems) using a touchdown program with an initial 15‐min denaturation at 95°C, followed by 2 cycles at 95°C for 30 s, annealing at 60°C for 30 s and extension at 72°C for 2 min. Every two cycles, the annealing temperature was reduced by 2°C (60–52°C) and then kept at 50°C for the remaining 30 cycles. Final extension occurred at 72°C for 5 min. Amplified products were purified using Exo‐SAP (New England Biolabs, MA, USA and Affymetrix, OH, USA) following the manufacturer's protocol. Sequencing of PCR products was performed using the two primers and the ABI PRISM BigDye Terminator cycle sequencing kit (Applied Biosystems) with an annealing temperature of 50°C. Cycle sequencing products were purified using Agencourt CleanSEQ Dye Terminator Removal (Beckman Coulter, CA, USA) and were run on an ABI 3130 Genetic Analyser (Applied Biosystems). Finally, sequences were aligned and edited using codoncode aligner v. 4.2.7 (CodonCode Corporation, https://www.codoncode.com).

### 2.3 Data analysis

#### 2.3.1 Sex‐biased dispersal

First, sex‐biased dispersal was investigated using the assignment test implemented in the program fstat v.2.9.3 (Goudet, Perrin, & Waser, 2002). The method assumes a species with nonoverlapping generations where dispersal occurs at the juvenile stage. As further postdispersal sampling is assumed, this test was conducted only on the nucDNA datasets of adult bats assumed to be postdispersal (*N*_F_ = 106, *N*_M_ = 196) using the sampling site as substructure. Expectations of this test are that the dispersing sex should show (1) greater variance in assignment (vAI_C_), (2) weaker source‐population‐assignment (mAI_C_), (3) higher within‐group diversity (*H*_S_), (4) a deficiency in heterozygotes due to samples representing a mixture of genetic populations (resulting in higher *F*_IS_‐values, showing signs of a Wahlund effect) and (5) lower diversity measures among groups (*F*_ST_) (Goudet et al., 2002). We used all the sets of tests mentioned above and conducted a one‐sided test, thereby either setting females or males to be the philopatric sex, respectively, with 10,000 permutations each.

To confirm the result achieved with all five measures by the test mentioned above (that males are the dispersing sex in *T. cirrhosus*, see Results), we conducted an additional run using the same tool in fstat v.2.9.3 (Goudet et al., 2002) to act as a control. This run was performed using males of different age groups, thereby treating juvenile (before their dispersal period) male bats as residents and adult male bats as the dispersers (*N*_ADULT_ = 196; *N*_JUVENILES_ = 60). As juvenile bats, identified by the presence of epiphyseal gaps in the phalanges (Brunet‐Rossini & Wilkinson, 2009), have not dispersed from their natal colony at the point they were sampled, their genetic constitution should categorize them as residents. Generally, very little is known about when juveniles leave their natal colony and differences between trophic groups have been described. In the phyllostomid frugivorous bat *Dermanura watsoni*, juveniles left the natal roost already at the age of 30–40 days (Chaverri & Kunz, 2006). However, in contrast to a bimodal reproductive phenology in frugivores, reproduction in animalivorous bats has been described to be unimodal (Durant, Hall, Cisneros, Hyland, & Willig, 2013). Thus, it can be expected that dispersing juveniles would leave their natal group before their mother gives birth to the next offspring (within the first year of their lives). Adult males, on the other hand, should have dispersed before being sampled and should therefore be categorized as dispersers. Again, a one‐sided test with 10,000 permutations was operated assuming juveniles to be residents (e.g., “philopatric”).

Further assessment of dispersal patterns was conducted by performing spatial autocorrelation analyses, that is, analyses of genetic relatedness between pairs of individuals as a function of the natural logarithm of geographical distance using SPAgedi 1.2 (Hardy & Vekemans, 2002) for two datasets separately. The first dataset consisted of adult females (data_F_) and the second one was comprised of all adult males (data_M_). The degree of spatial genetic structuring can be measured by the slope of the relationship mentioned above (Hardy & Vekemans, 2002). To obtain a multi‐allelic, multi‐locus mean measure of spatial genetic structure per given distance, the kinship coefficient *F*_*ij*_ presented in Loiselle, Sork, Nason, and Graham (1995) was estimated between all pairs of individuals. The kinship coefficient *F*_*ij*_ (Loiselle et al., 1995) was used as a pairwise estimator of genetic relatedness, as it represents a relatively unbiased estimator with low sampling variance. The standard error and significance of the linear regression slope were calculated by jackknifing (over loci) and by 10,000 permutations of locations. Moreover, the number of spatial distance categories was set to 10, and SPAgedi defined these 10 maximal distances ensuring that the number of pairwise comparisons within each distance interval was approximately constant. Significance of difference in slope between the two datasets was calculated using a permutation test in R (R Development Core Team, 2013), performing 1,000 randomizations (Frantz et al., 2010).

#### 2.3.2 Assessment of population genetic structuring on the basis of nucDNA

Before conducting population genetic structure analyses using the program structure (Pritchard, Stephens, & Donnelly, 2000), closely related individuals were removed from each dataset [(1) females + all juveniles irrespective of their sex (data_F+JUV_); (2) only adult males (data_M_)], as the presence of closely related individuals within populations can bias Bayesian multi‐locus clustering methods (Rodríguez‐Ramilo & Wang, 2012). Identification and removal of close relatives were performed following the procedure described in Halczok et al. (2017). Pairs of individuals whose relatedness value exceeded the determined threshold of 0.38 were identified and, consequently, one randomly chosen individual per pair was removed. This procedure was conducted for each sampling site separately. Therefore, potential close relatives encountered in different sampling sites were not impacted. Moreover, this was only performed on datasets intended for structure analyses, whereas all other analyses were performed on the whole dataset. The nucDNA dataset for females and all juveniles irrespective of their sex (data_F+JUV_) thereafter consisted of a total of 121 samples, and the dataset only consisting of adult males (data_M_) amounted to 142 samples (Table 2). The sampling site GI was excluded from structure analyses of data_M_ due to limited sample size (only one adult male).

**Table 2:** Number of samples used for structure for data_F+JUV_ (females + all juveniles irrespective of their sex) and data_M_ (only adult males) after the removal of close relatives

Sampling site	data_F+JUV_	data_M_
Barro Colorado Island (BCI)	12	23
Bohio (BO)	5	5
Gamboa (GA)	83^a^	97^a^
Gigante (GI)	6	—
Culebra Cut (CUL)	15	17

a Subsampling was carried out by randomly choosing 30 different individuals from this site and analysing 10 subsampled datasets.

structure was run on each nuclear DNA dataset assuming admixture and correlated allele frequencies using the LOCPRIOR model that allows for the use of sample group information (here the sampling sites BCI, BO, GA, GI, and CUL) in the clustering process (Hubisz, Falush, Stephens, & Pritchard, 2009). The LOCPRIOR model has been shown to detect genetic structure at lower levels of divergence, or with less data, than previous structure models, but does not tend to find structure where none is present (Hubisz et al., 2009). Ten independent runs of *K* = 1–10 were conducted for each of the two datasets, respectively. All runs used 10^6^ iterations after a burn‐in period of 10^5^.

As uneven sampling can bias inferences on the number of clusters in the program structure (Puechmaille, 2016), efforts were made to have comparable number of individuals from each sampling site after the removal of closely related individuals. Therefore, subsampling was carried out by randomly choosing 30 different individuals from GA (10 subsampled datasets were analyzed). Moreover, we followed the procedure from Puechmaille (2016) to ensure an accurate estimate of *K*. It has been recommended for low sample sizes in a priori defined groups (here: *N*_BO_ = 5 for each dataset) to set the threshold of mean membership coefficient in any subpopulation of the dataset to greater than 0.5 and to rather consider *MedMeaK* or *MedMedK* instead of *MaxMedK* or *MaxMeaK* (Puechmaille, 2016). Moreover, the larger a threshold value for the mean membership coefficient, the larger the differentiation between two subpopulations needs to be for them to be considered to belong to different genetic clusters. Therefore, setting a threshold value too high might potentially underestimate the real number of clusters. Therefore, we analyzed the results of the program structure using the thresholds 0.6, 0.7, and 0.8, but mainly focused on the threshold of 0.6 for the interpretation of the results.

For each of the genetically distinct populations inferred by structure (BCIGI, GABO, and CUL), the significance of deviations from Hardy–Weinberg equilibrium (HWE, Nei, 1978) was tested with the Markov‐chain method in genepop 4.0.7 (Raymond & Rousset, 1995) with 10,000 dememorization steps, 500 batches and 10,000 subsequent iterations. The same program was used to test the populations for linkage disequilibrium between loci, using an exact test based on a Markov‐chain method. The False Discovery Rate (FDR) correction technique was used to deal with multiple testing (Benjamini & Hochberg, 1995; Verhoeven, Simonsen, & McIntyre, 2005). Furthermore, microchecker 2.2.3 (Van Oosterhout, Hutchinson, Wills, & Shipley, 2004), set for 10,000 iterations and a 95% confidence interval, was used to test for null alleles. These analyses that are necessary to ensure that appropriate markers were used were only performed on residents, here data_F+JUV_. Population pairwise *F*_ST_ values (Wright, 1951) were used to measure the level of genetic differentiation for both datasets between the populations inferred by structure. *F*_ST_ values were calculated using fstat v.2.9.3.2 (Goudet, 1995) and significant difference from zero was tested with 10,000 permutations of individual genotypes between populations (Goudet, Raymond, deMeeus, & Rousset, 1996).

To assess the level of genetic diversity, the observed (*H*_o_) and expected heterozygosity (*H*_e_) for each locus as well as for each population inferred by structure for data_F+JUV_ were calculated using genetix 4.05.2 (Belkhir, Borsa, Chikhi, Raufaste, & Bonhomme, 1996). The mean number of alleles (*A*) and the allelic richness (*A*_R_), were calculated for each locus and each cluster using fstat v.2.9.3 (Table S1, supplementary material).

#### 2.3.3 Assessment of population genetic structuring on the basis of mtDNA

Regarding mtDNA, pairwise genetic distances were calculated between the 53 individual sequences using the Kimura 2‐parameter model implemented in mega 6.0 (Kimura, 1980; Tamura, Stecher, Peterson, Filipski, & Kumar, 2013). Additionally, a median‐joining (MJ) haplotype network was constructed using NETWORK v.5.0 (Bandelt, Forster, & Rohl, 1999) to graphically illustrate the relationships among the two different haplotypes found. Additionally, due to a lack of available sequences that span the complete fragment of the mitochondrial region that we investigated which also included noncoding and thus highly variable regions of the D‐loop, further sequences of only the cytochrome *b* of the mitochondrial genome of *T. cirrhosus* were obtained from GenBank [accession numbers: DQ233669 (Fonseca et al., 2007), DQ903828 and FJ155483 (Hoffmann, Hoofer, & Baker, 2008)] to set the haplotypes found in the course of this study into a larger context. For this comparison, the sequences obtained within this study were clipped to a length of 733 bp.

In addition to the haplotype network, phylogenetic reconstructions were performed using Bayesian inference in beast v1.7.4 (Drummond & Rambaut, 2007) under the GTR+I substitution model as determined by jmodeltest 2.1.7 (Darriba, Taboada, Doallo, & Posada, 2012; Guindon & Gascuel, 2003). beast was run for 10 million generations that were sampled in steps of 1,000, with a fixed substitution rate of 1.3 × 10^−8^ (Nabholz, Glemin, & Galtier, 2008; Puechmaille et al., 2012) and the constant size coalescent tree prior. No outgroup was specified. Results were visualized and interpreted using Figtree v1.4.3 (Rambaut, 2012).

Moreover, to estimate mtDNA divergence times, we used one individual of each of the two mitochondrial haplotypes determined, the three *T. cirrhosus* sequences from GenBank mentioned above, and sequences of the cytochrome *b* from two closely related bat species *(Lophostoma silvicolum* (HG003311) and *Tonatia saurophila* (HG003315); Botero‐Castro et al., 2013). For this purpose, phylogenetic reconstructions were performed under the same conditions as mentioned above.

#### 2.3.4 Assessment of capture rates

Additionally, capture rates were calculated for a subset of the individuals included in the genetic datasets mentioned above, as only those bats captured in accordance with the standardized mist‐netting approach (Meyer & Kalko, 2008a) were suitable for the calculation of capture rates (*N* = 29). This sampling took place between 2013 and 2016 within a total of 5380 mist netting hours (mnh). Capture rates were calculated for BCI, BO, GI, PB, all islands within the Panama Canal combined (I) and the forest fragments within the agriculturally managed land close to El Giral (A). Among others, the one individual sampled in A was not captured following the standardized mist‐netting approach and is therefore not represented here. We calculated the capture rate as a standardized measure of relative abundance by dividing the number of bats (recaptures included) captured at each sampling site by the number of mnh employed (1 mnh represents one 6 m mist net open for 1 hr). Relative abundance is then provided as number of bats per 100 mnh.

## 3 RESULTS

### 3.1 Sex‐biased dispersal

For the sex‐biased dispersal test between adult females and males assuming female philopatry, all five tests conducted in fstat were significant (*p* < .05) and clearly indicated male‐biased dispersal (Table 3). Additional tests under the assumption of males to be philopatric instead of females were all not significant and therefore indicated the same results of male dispersal and female philopatry (data not shown).

**Table 3:** Results of the one‐sided sex‐biased dispersal test conducted in fstat assuming female philopatry

	*N*	vAl_C_	mAl_C_	*H*_S_	*F*_IS_	*F*_ST_
Test for sex‐biased dispersal						
Adult females	106	11.8055	0.9386	0.6050	0.0558	0.0358
Adult males	196	46.2739	−0.5076	0.6280	0.0884	0.0201
*p*‐Value		.0259^a^	.0062^a^	.0030^a^	.0418^a^	.0099^a^
Additional test (males of different age groups)						
Juvenile males	60	14.7684	0.9815	0.6118	0.0761	0.0620
Adult males	196	44.2519	−0.3005	0.6280	0.0884	0.0201
*p*‐Value		.3748	.0538	.0442^a^	.1887	.0038^a^

Calculated values for variance in assignment (vAI_C_), mean source‐population‐assignment (mAI_C_), within‐group diversity (*H*_S_), the inbreeding coefficient of an individual relative to the group (*F*_IS_) and diversity measures among groups (*F*_ST_) are listed together with their appertaining *p*‐values.

a Significant *p*‐values at the .05 nominal level.

The additional test between males of different age groups to further confirm the determined male‐biased dispersal showed comparable results. For this test, the difference in *F*_ST_ and *H*_S_ were significant (*p* < .05, Table 3). For all the other measures, a nonsignificant trend for adult males being the dispersing sex is visible (Table 3). Additional tests under the assumption of adult males to be residents were not significant and therefore indicated the same results (data not shown).

**Figure 2:**
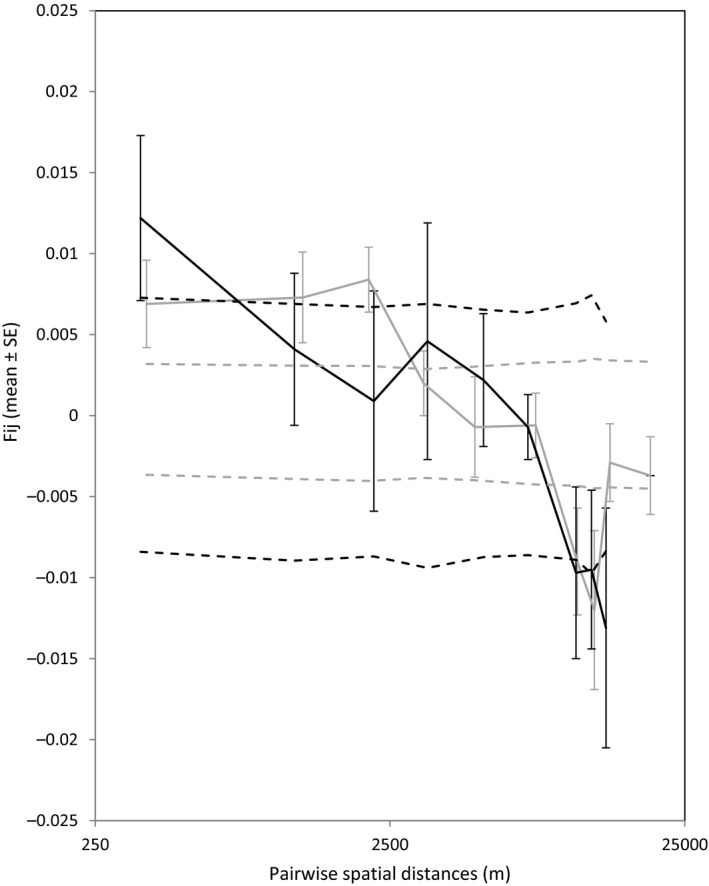
Average kinship coefficients, *F*_*ij*_, between pairs of individuals plotted against geographical distance for data_F_ (adult females) displayed in black and data_M_ (adult males) displayed in gray. The dashed lines represent 95% confidence intervals for *F*_*ij*_ under the null hypothesis that genotypes are randomly distributed

When regressing geographic distance and relatedness between individuals belonging to respective datasets, weak but significant regression patterns of isolation‐by‐distance were detected (slope *b*_F_ ± *SE* = −0.0047 ± 0.00097, *p* = .0005; slope *b*_M_ ± *SE* = −0.0038 ± 0.00043, *p* < .0001; Figure 2). The increase in genetic differentiation among individuals with geographical distance is more pronounced in data_F_ as indicated by a steeper slope compared with data_M_. However, this difference in slope was not statistically significant (*p* = .274).

### 3.2 Assessment of population genetic structuring on the basis of nucDNA

**Figure 3:**
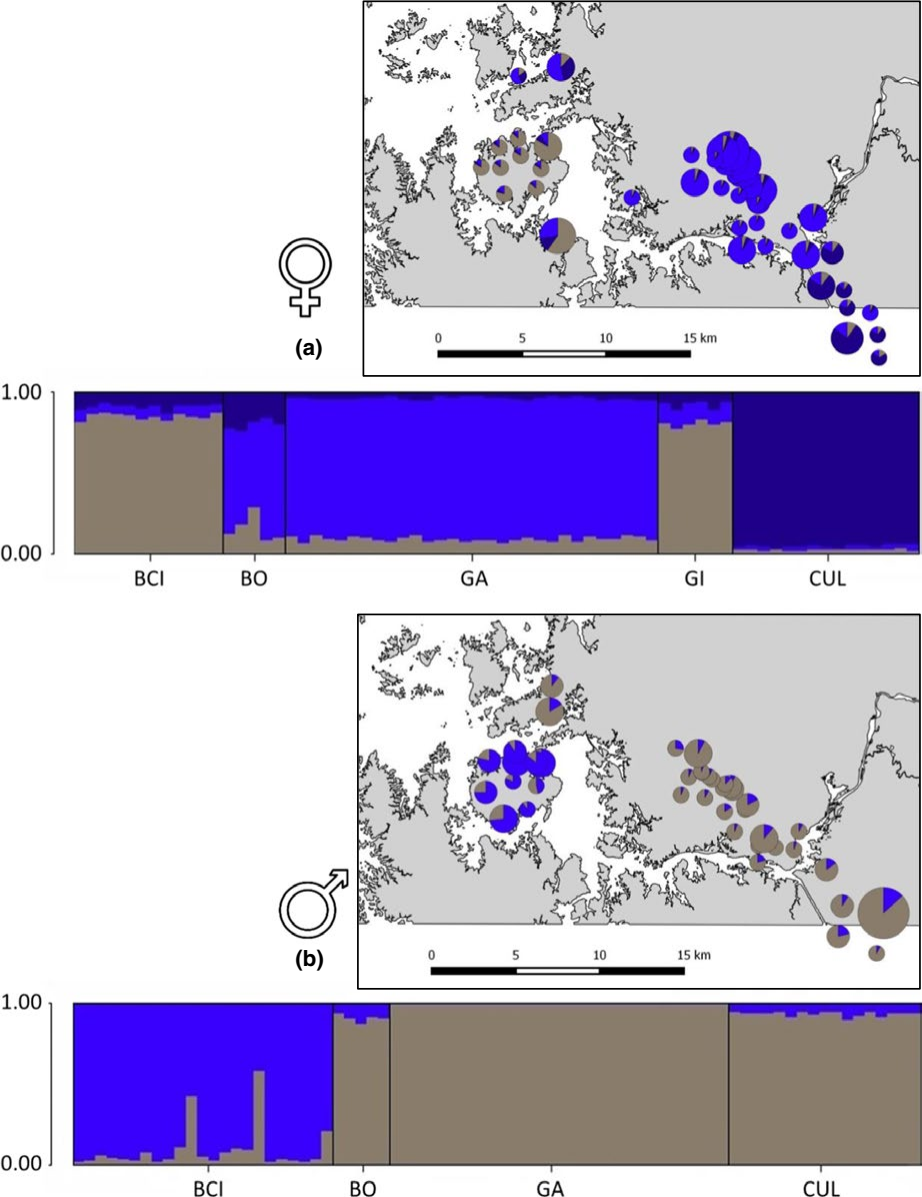
Bar plot graph of estimated membership coefficient of *Trachops cirrhosus* from Bayesian analysis generated using structure and the locprior option for (a) data_F+JUV_ (females + all juveniles irrespective of their sex; *K *=* *3) and (b) data_M_ (only adult males; *K *=* *2). BCI, Barro Colorado Island; BO, Bohio; GA, Gamboa; GI, Gigante; CUL, Culebra Cut. Additionally, the maps display an overview of the netting sites and the cluster assignment of the sampled individuals. Sizes in the map are proportional to sample size

Using the 16 autosomal microsatellite loci, the Bayesian clustering method inferred three distinct genetic clusters for data_F+JUV_ (Figure 3a) based on the estimators *MedMeaK* and *MedMedK* for a threshold of 0.6 and two genetic clusters for threshold values of 0.7 and 0.8 (Figure S1a, supplementary material). For data_M_, for the threshold value of 0.6, both estimators clearly point toward *K *=* *2, whereas for 0.7 and 0.8 *MedMeaK* and *MedMedK* rather point toward a *K* of 1 (Figure S1b, supplementary material). When comparing the results for data_F+JUV_ and for data_M_ at the same threshold value, we can clearly see that the amount of genetic structuring differs between the sexes, with males showing less genetic structuring than females and juveniles.

Taking all of these considerations into account, for data_F+JUV,_ *K *=* *3 shows the strongest support, whereas BCI clusters with GI (BCIGI_F+JUV_), GA with BO (GABO_F+JUV_) and CUL represents the third cluster (CUL_F+JUV_). For data_M_, *K *=* *2 shows the clearest signal, whereas BCI represented one cluster and all other sites were encompassed in the second one (Figure 3b).

When testing each of the three populations inferred by the program structure for data_F+JUV_ for HWE no significant deviations were detected. Furthermore, no deviations from linkage disequilibrium at the α = 0.05 level occurred after FDR correction. Finally, no marker showed consistent evidence for the presence of null alleles.

Analysing data_F+JUV_ using the program fstat v.2.9.3.2 (Goudet, 1995), we detected significant pairwise genetic differentiation between the three genetic clusters identified by structure (*F*_ST_(BCIGI_F+JUV_/GABO_F+JUV_) = 0.0392, *p* = .0167; *F*_ST_(BCIGI_F+JUV_/CUL_F+JUV_) = 0.0440, *p* = .0167; *F*_ST_(GABO_F+JUV_/CUL_F+JUV_) = 0.0417, *p* = .0167). As for data_M_ *F*_ST_ was calculated between BCI and the combined other sampling sites where adult males occurred. Here, *F*_ST_ value was also significant (*F*_ST_ = 0.0247, *p* = .05).

### 3.3 Assessment of population genetic structuring on the basis of mtDNA

**Figure 4:**
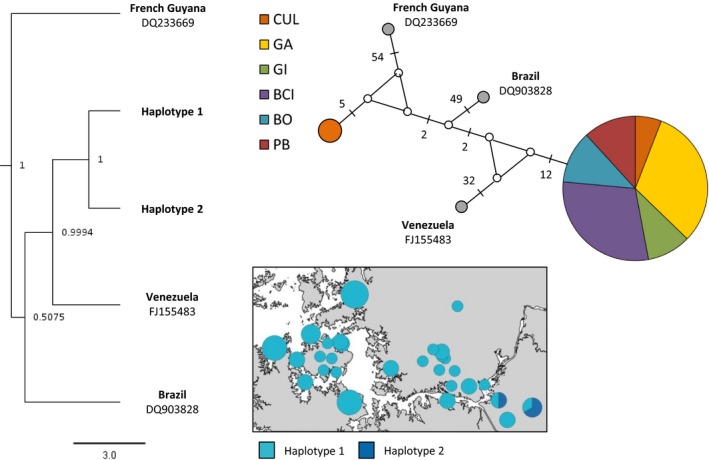
Posterior phylogenetic tree and median‐joining haplotype network for *Trachops cirrhosus* based on 733 bp of mtDNA (cytb). Sampling sites (CUL, Culebra Cut; GA, Gamboa; GI, Gigante; BCI, Barro Colorado Island; BO, Bohio; PB, Peña Blanca) and haplotype frequency scale are shown in the inset. All mtDNA sequences (*n* = 53) were used in this Median‐joining network. Branch lengths are not proportional to base‐pair changes (all changes are 1 base pair unless otherwise indicated). Additionally, the map displays an overview of the netting sites and the haplotypes found. Sizes in the map are proportional to sample size

Regarding mtDNA, the 53 sequences were successfully aligned resulting in a 1,605 bp long fragment. Only two different haplotypes were found within the study area with a K2P genetic distance of 2.83% (GenBank accession numbers: MH102398, MH102399). Fifty individuals represented haplotype 1, and three individuals, all sampled in CUL, displayed haplotype 2. Throughout the complete fragment of mtDNA analyzed, the two haplotypes differed from each other at 46 sites. Within the cytochrome *b* alone, the two discovered haplotypes still differed at 23 sites (Figure 4). Moreover, setting them in context with sequences derived from GenBank that originate from French Guyana, Venezuela, and Brazil shows that even though these two sequences are still most similar to each other, they fit well into context with samples from other geographical regions within Central/South America (Figure 4).

The time of divergence between the two mitochondrial haplotypes found in the course of this study was estimated to have occurred 1.3 million years ago. Highest posterior density for the time to the most recent common ancestor was estimated to be between 766,000 and 1,870,400 years ago.

### 3.4 Assessment of capture rates

According to the capture rates obtained for individuals sampled during the standardized mist‐netting approach, the relative abundance of *T. cirrhosus* was much lower in the two highly fragmented areas *A* (0.00) and *I* (0.04) compared to the other sites that were mainly dominated by continuous rainforest (BCI = 1.20; BO = 1.92; GI = 2.78; PB = 1.39).

## 4 DISCUSSION

Several lines of evidence clearly indicate that *T. cirrhosus* shows a pattern of male‐biased dispersal. In terms of gene flow, male‐biased dispersal has various consequences and can thus be identified by comparing the sex‐specific population genetic patterns of a species (Freeland, Kirk, & Petersen, 2011a; Prugnolle & de Meeus, 2002). First, it has been hypothesized that the dispersing sex should show lower levels of among‐population differentiation than the philopatric sex (e.g., Mossman & Waser, 1999). Indeed, our results demonstrate that males show lower levels of population differentiation. This is indicated by only two genetic clusters in the male dataset compared to the three distinct genetic clusters identified in data_F+JUV_, and by lower *F*_ST_ values between genetic clusters in data_M_ (0.0247), compared to data_F+JUV_ (0.0392–0.0440). Secondly, spatial autocorrelation, or the decrease in genetic relatedness among individuals with geographical distance (e.g., Frantz et al., 2010), in *T. cirrhosus* is more pronounced in data_F_ as indicated by a steeper slope compared with data_M_. Even though these slopes of isolation‐by‐distance do not differ significantly between the sexes, a trend is visible. Third, assignment tests can be used to compare the number of individuals that are genetically assigned to a population different to the one where they were sampled in. Consistent with the assumption that the dispersing sex should show significantly more miss‐assignments (Goudet et al., 2002), male *T. cirrhosus* showed both a significantly greater variance in assignment and a weaker source‐population‐assignment compared to females which clearly indicates male‐biased dispersal. Finally, in the case of male‐biased dispersal, maternally inherited mtDNA markers should show higher levels of population differentiation compared to biparentally inherited markers. Unfortunately, within our study area, we only detected two mitochondrial haplotypes. Although these haplotypes did differ strongly from each other (genetic distance = 0.0283), one of the haplotypes was only found in three of six individuals from CUL and all other individuals showed the same mitochondrial haplotype. Therefore, no conclusions can be drawn here in terms of sex‐specific dispersal.

The presence of only one dominant mitochondrial haplotype within the study area is quite surprising for tropical species (compare Ditchfield, 2000). In particular, as our mtDNA sequences comprise coding regions, such as the full cytochrome *b*, as well as noncoding parts of the D‐loop region, which is well known for its high mutation rate (e.g., Wilkinson, Mayer, Kerth, & Petri, 1997). The fact that the full cytochrome *b* sections of our sequences did not harbor any stop codon suggests amplification of the true mtDNA rather than nuclear copies, known as Numts (Triant & Dewoody, 2007). This remarkably low level of mitochondrial diversity in *T. cirrhosus* could be related to the history of the species in the last million years, as the divergence of the two strongly differentiated mitochondrial haplotypes (approximately 3% differentiation) was estimated to have occurred in the early Pleistocene (between 766,000 and 1,870,400 years ago). However, due to a lack of a more complete geographic sampling across the species range, we can only speculate about the origin of these distinct mtDNA haplotypes.

Critically, if our study had only focused on the analysis of mtDNA, the lack of spatial differentiation may have led to the tentative conclusion that females are the dispersing sex. Therefore, our study emphasizes that it is not always sufficiently conclusive to compare the results of uni‐parentally and biparentally inherited markers to make inferences about sex‐specific dispersal, but additionally to search for signs of sex‐specific dispersal among the biparentally inherited genotypes (Goudet et al., 2002).

Male‐biased dispersal is the typical pattern for temperate zone bat species (see Moussy et al., 2013 for a review). In Neotropical bats, however, various patterns of natal dispersal have been described with male‐biased dispersal being comparatively rare (e.g., Dechmann et al., 2007; Nagy, Gunther, Knornschild, & Mayer, 2013; Wilkinson, 1985). Generally, philopatry is assumed to be the optimal strategy for female mammals due to the various benefits accrued by the females staying in or in close proximity to the natal area and/or social group (Greenwood, 1980). These benefits include familiarity with local resources, and improved fecundity and breeding success when associating with kin (e.g., Clutton‐Brock & Lukas, 2012). Despite this general assumption, Nagy et al. (2013) hypothesized that a large number of Neotropical bat species have dispersal patterns that differ from those of the majority of mammals. This hypothesis was based on the fact that female breeding behavior in Neotropical bats (especially in some phyllostomid bats) has been shown to commence very early in life (e.g., in *Dermanura watsoni* sexual maturity of females was reached in as little as 3 months; Chaverri & Kunz, 2006). Female dispersal allows females to avoid father‐daughter inbreeding if the age of females at first conception falls below the tenure of males (Dechmann et al., 2007).

Female *T. cirrhosus* have been observed to be lactating within less than a year after being captured as a juvenile (V. Flores, unpublished data). We can therefore assume that this species reaches sexual maturity as early as other phyllostomids (Chaverri & Kunz, 2006). Consequently, one would expect female dispersal in *T. cirrhosus* as well. However, this conclusion might be immature because Nagy and co‐workers discovered a male‐biased dispersal pattern within *Balantiopteryx plicata* (Nagy, Knornschild, Gunther, & Mayer, 2014), a bat species where the age of females at first conception fell below the tenure of males. They argue that the reason for this was that father–daughter inbreeding was circumvented by mating outside of the nursing roost.

In phyllostomid bats, natal dispersal has thus far only been investigated in four species. In *Uroderma bilobatum* habitat characteristics, specifically, the characteristics of their roosts, seem to determine whether females disperse or show philopatry (Sagot, Phillips, Baker, & Stevens, 2016). Both *Lophostoma silvicolum* (Dechmann et al., 2007) and *Phyllostomus hastatus* (McCracken & Bradbury, 1981) show all‐offspring dispersal, which seems to have developed due to a mixture of inbreeding avoidance and avoidance of local mate competition and local resource competition. In Central Panama, female *T. cirrhosus* might profit from knowing their local habitat because this species’ relatively small foraging grounds (Jones et al., 2017; Kalko et al., 1999) mainly consist of streams and ponds, which are patchily distributed and thus harder to find. Therefore, females might profit from staying in or at least close to their natal habitat. Note, that with our method, we cannot discriminate between strict philopatry where females stay in the natal area/group and philopatry that results from females moving much shorter distances than males. However, unpublished capture‐mark‐recapture data suggest that at least some females remain in their natal colony.

In *Desmodus rotundus*, dispersal is male‐biased (Streicker et al., 2016; Wilkinson, 1985). As cooperation both between related and unrelated females is frequently observed in this species (Carter & Wilkinson, 2013), (kin)cooperation seems to be the driving force for the observed female philopatry and male‐biased dispersal. While *T. cirrhosus* can quickly acquire novel foraging behavior by observing conspecifics (Jones, Ryan, Flores, & Page, 2013; Page & Ryan, 2006), the social learning observed in this species is via eavesdropping where one individual opportunistically observes the foraging behavior of another. Thus, unlike *Desmodus rotundus*, female *T. cirrhosus* are likely not cooperating while foraging or in sharing food resources at the roost. Therefore, female cooperation as the force driving male‐biased dispersal seems unlikely.

Generally, female dispersal patterns are primarily determined by resource distribution, while the dispersal patterns of males are mainly influenced by female dispersal (Clutton‐Brock & Lukas, 2012). As females typically tend to avoid inbreeding, males need to be the dispersing sex when females show restricted dispersal (e.g., Clutton‐Brock & Lukas, 2012). Consequently, avoidance of inbreeding, coupled with local mate competition and local resource competition, should be considered as the potential evolutionary forces responsible for male‐biased dispersal in *T. cirrhosus*.

In addition to the male‐biased dispersal pattern discovered, the results of this study further indicate the presence of population genetic structuring. The fact that BCI is genetically separated from the other populations studied (GABO and CUL), even within the male dataset, suggests that gene flow is to a certain extent impeded within our study area. If the former Río Chagres (Figure 1) had impeded gene flow, CUL should be genetically distinct from GA in both datasets. While this differentiation is present in the dataset consisting of females and juveniles, this is not the case for males. Moreover, the former Río Chagres would not have been a barrier between BCIGI and CUL because it did not separate those sites geographically, but the Panama Canal now does. Starting from Gamboa, the Panama Canal does not follow the route of the historical river anymore but continues on toward Panama City and ultimately to the Pacific Ocean. While the maximum width of continuous water body of the part of the Río Chagres, which was not changed during the construction of the Panama Canal amounts to approximately 730 m close to Gamboa, approximately 2.5 km upstream it becomes narrower with a maximum width of about 130 m. In contrast, the narrowest part of the Panama Canal within our study area, the Culebra Cut, is approximately 250 m in width. Unfortunately, no data are available for the former width of the currently flooded part of the Río Chagres.

In this context, it is not surprising that bats can cross between BCI and GI even though due to the flooding of the historical river water is also separating BCI from the Southern mainland side of the Panama Canal. Between BCI and the GI side of the mainland, there are various islands that can be used as stepping stones. The largest continuous water body a bat necessarily has to cross here only amounts to 125 m in the southwest of BCI thereby reaching an island that is only separated from the mainland by 110 m. These distances seem to be traversable for *T. cirrhosus* as we find no evidence for genetic structuring between BCI and the Southern side of the Panama Canal, including GI.

Overall, our results suggest that the human‐induced increase in width of a former water body between BCIGI and the Northern mainland side of the Panama Canal poses an impediment to gene flow in *T. cirrhosus*. This would suggest that, even within a very short time interval of approximately 100 years, changes in population genetic patterns can be observed within a bat species (consistent with Meyer et al., 2009). However, due to limited sample sizes and sampling sites in this study, further exploration on the potential presence of this relatively new, human‐induced impediment to gene flow are necessary.

In addition to the potential introduction of barriers to dispersal, human‐induced habitat fragmentation may also critically limit the distribution of *T. cirrhosus* as a function of reduced population viability in smaller habitat fragments. This is supported by the observation that *T. cirrhosus* was not caught in the highly fragmented, agriculturally dominated landscape around El Giral (Figure 1) on the smaller islands within the Panama Canal (results consistent with former studies: Meyer & Kalko, 2008b; Meyer et al., 2009). The capture rates determined in the course of this study clearly show that the relative abundance of *T. cirrhosus* was much lower in *A* (0.00) and *I* (0.04) compared to the other sites (BCI = 1.20; BO = 1.92; GI = 2.78; PB = 1.39). This further emphasizes the species’ sensitivity and vulnerability to fragmentation (as predicted by Kalko et al., 1999).

Mobility has been suggested to be a good predictor of a species’ vulnerability to fragmentation and altered population genetic structure. In bats, the ability and proclivity for long distance flight and dispersal is mostly reflected in the wing morphology of a species (Meyer et al., 2009). *Trachops cirrhosus* has short, broad wings which allow it to be highly maneuverable in obstacle‐rich environments (Norberg & Rayner, 1987). This low wing loading is efficient for gleaning prey in the forest understory, but means that *T. cirrhosus* is a slow flyer. Both GPS (S. Greif, unpublished data) and telemetry tracking studies (Jones et al., 2017; Kalko et al., 1999) show that *T. cirrhosus* moves very little within the landscape, mostly stays within the forest understory, and tends to avoid open areas. In accordance with these findings, our own observations and genetic results confirm the classification of *T. cirrhosus* as a less mobile species (Meyer et al., 2009).

## 5 CONCLUSIONS

In accordance with our hypothesis, our results indicate that habitat fragmentation seems to influence both the population genetic structure and the distribution of *T. cirrhosus*. Moreover, we were able to demonstrate that *T. cirrhosus* shows male‐biased dispersal. Further research is needed to investigate the driving force for the strong pattern of male‐biased dispersal observed in *T. cirrhosus*.

Finally, the effect of the Panama Canal as a potential barrier to gene flow should be further investigated in *T. cirrhosus* as well as in other bat species within the same habitat. In this context, the phyllostomid family would be particularly interesting as species of this family are relatively closely related, but show high levels of ecological diversity through various differences in morphological, physiological and behavioral traits (Baker, Jones, & Carter, 1976; Datzmann, von Helversen, & Mayer, 2010). Therefore, the same fragmented habitat might appear to hinder movement for one species, thus impeding genetic connectivity, while for another closely related species with a different ecology, it may allow uninhibited movements resulting in no genetic structuring.

This is the first study to provide population genetic data for *T. cirrhosus*. Knowledge on sex‐specific patterns of natal dispersal is important for understanding the social system of this otherwise well‐studied species and can be used as a basis for future social and behavioral investigations. Moreover, the results from our study may be used to inform effective conservation measures in regions suffering from a high impact of human‐induced habitat fragmentation and to gain additional knowledge on the effects of environmental disturbance on bats.

## CONFLICT OF INTEREST

The authors declare that they have no conflict of interest.

## AUTHOR CONTRIBUTIONS

TH and GK designed the study with input from RP and SJP. RP, SB, MT, VF, and TH provided data. TH performed the laboratory work and the data analyses and drafted the manuscript with input from RP, SJP, and GK. SJP contributed further to analyses. SB conducted the analyses on the capture rates at the different sampling sites. All authors have commented on the manuscript and have read and approved the final version of the manuscript.
