# Supplementary figures and images for: Male‐biased dispersal and the potential impact of human‐induced habitat modifications on the Neotropical bat Trachops cirrhosus
Source: Ecol Evol. 2018 May 15;8(12):6065–80. doi: 10.1002/ece3.4161 (PMC6024115; doi:10.1002/ece3.4161)

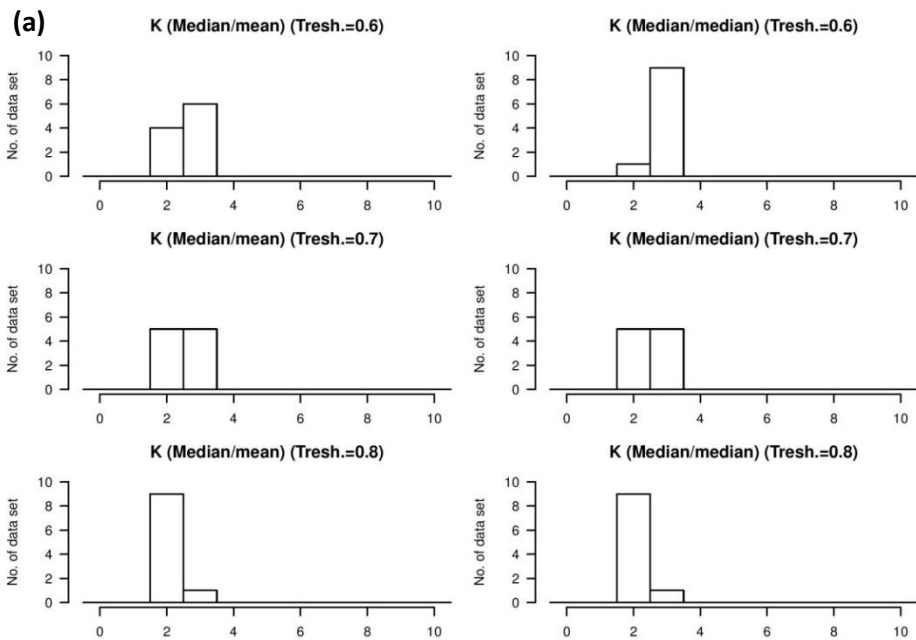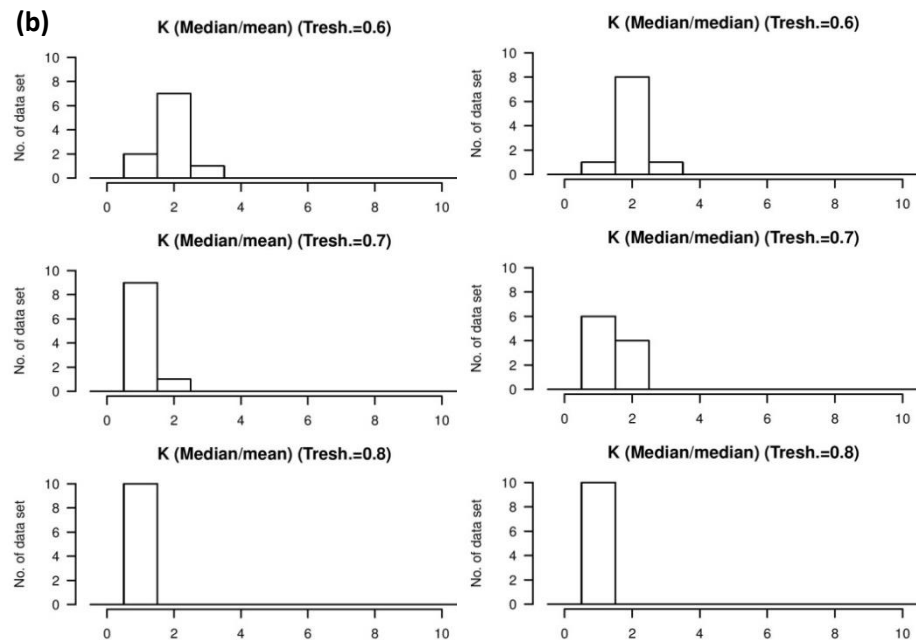

Supplement: Supplementary file 1 [file ECE3-8-6065-s001.pdf]
