# Supplementary material for: Male‐biased dispersal and the potential impact of human‐induced habitat modifications on the Neotropical bat Trachops cirrhosus
Source: Ecol Evol. 2018 May 15;8(12):6065–80. doi: 10.1002/ece3.4161 (PMC6024115; doi:10.1002/ece3.4161)
Supplement: Supplementary file 2 [file ECE3-8-6065-s002.docx]

**Supplementary Information**

**Table S1** Genetic diversity for *T. cirrhosus* at nuclear loci within genetic clusters inferred by Structure for the dataset consisting of females and juveniles of both sexes (data_F+juv_)

|  | ***Final concentration*** | ***BCIGI*** | | | | |  | ***GABO*** | | | | |  | ***CUL*** | | | | |
| --- | --- | --- | --- | --- | --- | --- | --- | --- | --- | --- | --- | --- | --- | --- | --- | --- | --- | --- |
|  | ***[µM]*** | *N* | *A* | *H_e_* | *H_o_* | *A_r_* |  | *N* | *A* | *H_e_* | *H_o_* | *A_r_* |  | *N* | *A* | *H_e_* | *H_o_* | *A_r_* |
| ***Multiplex 1*** |  |  |  |  |  |  |  |  |  |  |  |  |  |  |  |  |  |  |
| *Tcir2* | 0.50 | 19 | 5 | 0.6898 | 0.7368 | 4.7890 |  | 144 | 7 | 0.6938 | 0.6736 | 5.0770 |  | 21 | 4 | 0.6621 | 0.7143 | 3.8100 |
| *Tcir5* | 0.30 | 17 | 8 | 0.8512 | 0.7647 | 8.0000 |  | 143 | 16 | 0.8864 | 0.8951 | 10.9160 |  | 21 | 10 | 0.8299 | 0.7619 | 9.3580 |
| *Tcir9* | 1.10 | 19 | 6 | 0.6454 | 0.5789 | 5.8860 |  | 144 | 6 | 0.7078 | 0.7153 | 5.3040 |  | 21 | 6 | 0.7732 | 0.9048 | 5.9940 |
| *Tcir11* | 0.60 | 19 | 5 | 0.7202 | 0.4737 | 5.0000 |  | 143 | 5 | 0.7381 | 0.6434 | 4.7200 |  | 19 | 5 | 0.6690 | 0.5263 | 4.8940 |
| *Tcir13* | 1.00 | 18 | 4 | 0.5725 | 0.2778 | 3.9980 |  | 132 | 5 | 0.5189 | 0.3258 | 3.9870 |  | 20 | 3 | 0.2963 | 0.2500 | 2.8500 |
| *Tcir28* | 0.20 | 19 | 6 | 0.7202 | 0.7368 | 5.8860 |  | 144 | 8 | 0.7435 | 0.7292 | 6.1500 |  | 21 | 6 | 0.7891 | 0.6667 | 5.9900 |
| *Tcir40* | 0.16 | 19 | 2 | 0.0512 | 0.0526 | 1.8950 |  | 144 | 3 | 0.4106 | 0.2986 | 2.9850 |  | 21 | 3 | 0.2846 | 0.2381 | 2.8100 |
|  |  |  |  |  |  |  |  |  |  |  |  |  |  |  |  |  |  |  |
| ***Multiplex 2*** |  |  |  |  |  |  |  |  |  |  |  |  |  |  |  |  |  |  |
| *Tcir12* | 0.04 | 19 | 5 | 0.7133 | 0.6316 | 5.0000 |  | 144 | 8 | 0.5606 | 0.5278 | 5.9730 |  | 21 | 4 | 0.4524 | 0.4762 | 3.8090 |
| *Tcir20* | 0.10 | 19 | 5 | 0.7535 | 0.9474 | 4.8950 |  | 144 | 6 | 0.6341 | 0.6736 | 4.9530 |  | 21 | 5 | 0.7041 | 0.8571 | 4.8090 |
| *Tcir22* | 0.12 | 19 | 6 | 0.7064 | 0.6316 | 5.8940 |  | 144 | 7 | 0.7675 | 0.7222 | 5.6070 |  | 21 | 6 | 0.6893 | 0.7619 | 5.6180 |
| *Tcir24* | 0.09 | 19 | 5 | 0.7341 | 0.6316 | 5.0000 |  | 144 | 5 | 0.6264 | 0.6389 | 4.4380 |  | 21 | 5 | 0.6066 | 0.6190 | 4.7770 |
| *Tcir25* | 0.60 | 19 | 1 | 0.0000 | 0.0000 | 1.0000 |  | 143 | 2 | 0.4233 | 0.3007 | 2.0000 |  | 21 | 2 | 0.0907 | 0.0952 | 1.9670 |
| *Tcir26* | 0.04 | 19 | 3 | 0.2341 | 0.2632 | 2.8950 |  | 144 | 3 | 0.1538 | 0.1667 | 2.1660 |  | 21 | 2 | 0.2449 | 0.1905 | 2.0000 |
| *Tcir35* | 0.02 | 19 | 4 | 0.5720 | 0.4737 | 3.9830 |  | 143 | 4 | 0.5815 | 0.5594 | 3.9790 |  | 21 | 4 | 0.6485 | 0.7619 | 3.9670 |
| *Tcir38* | 0.08 | 19 | 5 | 0.6330 | 0.4737 | 4.8950 |  | 141 | 5 | 0.5511 | 0.4255 | 3.6920 |  | 21 | 3 | 0.6020 | 0.6667 | 3.0000 |
| *Tcir39* | 0.10 | 19 | 8 | 0.8296 | 0.9474 | 7.8850 |  | 142 | 10 | 0.8486 | 0.8592 | 8.2450 |  | 21 | 8 | 0.7494 | 0.7619 | 7.5850 |

*N* number of samples successfully analysed (total number of individuals: Barro Colorado Island + Gigante (BCIGI) 19, Gamboa + Bohio (GABO) 144, Culebra Cut (CUL) 21); *A* number of alleles; *H_o_* observed heterozygosity; *H_e_* expected heterozygosity; *A_R_* allelic richness based on a minimum of 19 individuals.

**Figure S1** Estimated values of *K* for (a) data_F+juv_ (females + all juveniles irrespective of their sex) and (b) data_M_ (only adult males) using MedMeaK and MedMedK (Puechmaille, 2016). Estimators were computed for different thresholds at which a subpopulation was considered to belong to a cluster; threshold noted in the title of each graph.
